# Diagnostic Significance of Absence of Post-Feeding Contraction of the Gallbladder in Biliary Atresia: Two Case Reports

**DOI:** 10.3390/pediatric15030049

**Published:** 2023-09-06

**Authors:** Masahiko Kosuga, Yoshimitsu Fujii, Takashi Doi, Kazunari Kaneko, Raoul Breugelmans

**Affiliations:** 1 Department of Pediatrics, Kansai Medical University, 2-5-1 Shin-machi, Hirakata 573-1010, Osaka, Japan; kosugam@hirakata.kmu.ac.jp (M.K.);; 2 Department of Pediatric Surgery, Kansai Medical University, 2-5-1 Shin-machi, Hirakata 573-1010, Osaka, Japan; 3 Department of English, Kansai Medical University, 2-5-1 Shin-machi, Hirakata 573-1010, Osaka, Japan

**Keywords:** biliary atresia, contraction of gallbladder, feeding, pancreaticobiliary maljunction, triangular cord sign, ultrasonography

## Abstract

Ultrasonography is an essential part of the diagnostic process of biliary atresia (BA). The characteristic findings of BA include a hilar hyperechoic zone, the triangular cord sign (TCS), an absence of gallbladder contraction after feeding, and gallbladder atrophy. However, approximately 10% of patients with BA have a normal gallbladder. We herein present two cases of BA with normal morphology of the gallbladder as shown by ultrasonography. In the first case, the patient was positive for the TCS, negative for gallbladder atrophy, and positive for contraction of the gallbladder after feeding; the final diagnosis was hilar obstructive BA complicated by pancreaticobiliary maljunction. In the second case, the patient was positive for the TCS, negative for gallbladder atrophy, and negative for contraction of the gallbladder after feeding; the patient also had common bile duct obstruction and stenosis of the hepatic duct in the hilar region. Based on these two cases, we conclude that gallbladder findings are not diagnostic for BA because in some types, the gallbladder may be normal in morphology and even have the capacity for contraction after feeding.

## 1. Introduction

Biliary atresia (BA) is a neonatal disease characterized by obstructive jaundice secondary to obstruction of the extrahepatic bile duct, and it occurs in 1 in 10,000 live births. Abdominal ultrasonography is often the first step in the evaluation of patients with persistent jaundice. Ultrasonographic findings of BA include the triangular cord sign (TCS) in the hilar region and a small or unidentified gallbladder [1,2]. However, a normal gallbladder with an open lumen is present in approximately 10% of cases [3]. One of the mechanisms of BA in patients with a normal gallbladder is pancreaticobiliary maljunction (PM), in which the common bile duct is patent and the contents of the gallbladder are composed of refluxed pancreatic fluid [4]. Thus, there are various forms of BA [5], and the ultrasonographic findings of the gallbladder may differ depending on the type of BA. However, there are few detailed reports on this topic. In this study, we present a discussion of the pathogenesis of two different types of BA with normal morphology of the gallbladder.

## 2. Cases

### 2.1. Case 1: 4-Day-Old Boy

The pregnancy was established by assisted reproductive technology and was uneventful throughout its course. Vaginal delivery occurred at 38 weeks and 5 days of gestation. The birth weight was 3164 g, and the body length was 49.5 cm. At 3 days of age, the serum bilirubin level was 150 mg/L, and 24 h phototherapy was administered; however, the total bilirubin level did not decrease. Grayish-white stools were also observed at the same time, and the patient was referred to our clinic at 4 days of age.

**Figure 1:**
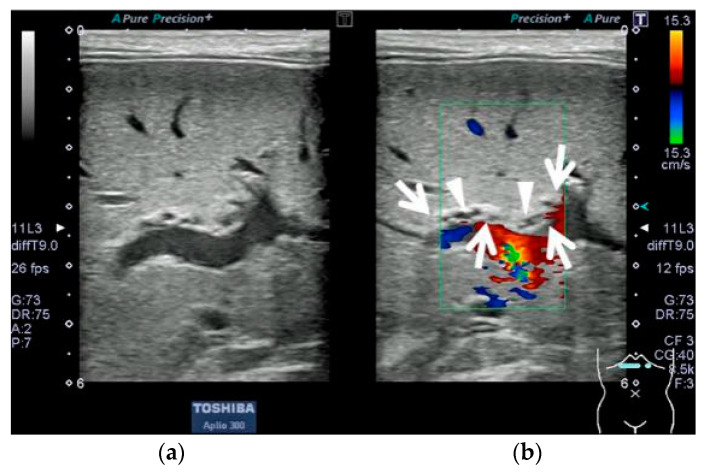
Abdominal ultrasonographic findings in Case 1 after 3 h of fasting; Aplio 300 (Cannon Medical Systems), linear probe, B-mode frequency: 9.0 MHz, pulse repetition frequency: 12 fps. (**a**) Transverse section in the right upper abdominal region, B-mode imaging at the porta hepatis. (**b**) Color Doppler imaging in the same section as (**a**): A hyperechoic mass was observed at the porta hepatis (enclosed by arrows). Internal luminal and microcystic structures without a blood flow signal were present on Doppler imaging (arrowheads).

Blood biochemistry tests at the initial visit revealed obstructive jaundice (aspartate aminotransferase, 32 U/L (reference range, 20–62 U/L); alanine aminotransferase, 14 U/L (reference range, 11–45 U/L); total bilirubin, 160 mg/L (reference range, <20 mg/L); direct bilirubin, 16 mg/L (reference range, <3 mg/L); gamma-glutamyl transpeptidase, 1003 U/L (reference range, 50–350 U/L); serum pancreatic amylase, 1 U/L (reference range, 1–6 U/L); and total bile acids, 612 µmol/L (reference range, <20 µmol/L)). Abdominal ultrasonography showed the TCS with internal luminal and microcystic structures and a dilated gallbladder lumen. The gallbladder wall was 1.0 mm thick and exhibited a two-layered appearance, and there was no evidence of gallbladder atrophy (Figure 1).

**Figure 2:**
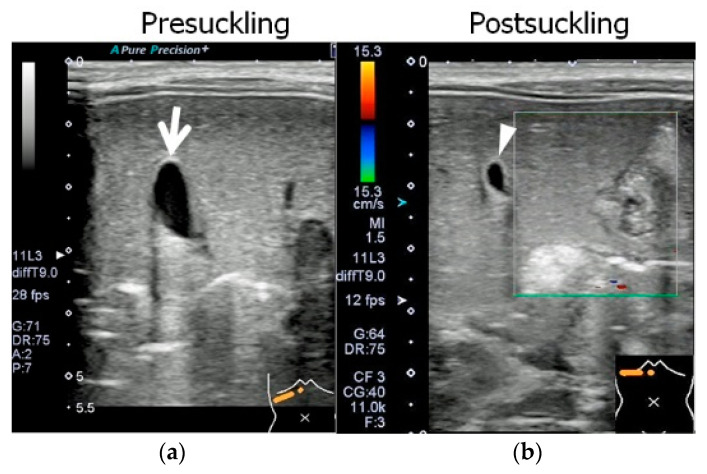
Abdominal ultrasonographic findings in Case 1 before and after feeding; Aplio 300 (Cannon Medical Systems), linear probe, B-mode frequency: 9.0 MHz, pulse repetition frequency: 12 fps. Images are presented in the right upper abdominal region, on the same scale as in Figure 1. (**a**) The gallbladder was examined at the same time as in Figure 1. The gallbladder lumen (arrow) was open. The measurements of the gallbladder were a long diameter of 18.0 mm, short diameter of 7.2 mm, and lumenal cross-sectional area of 0.53 cm^2^. The gallbladder wall, which was 1.0 mm thick and exhibited a two-layered appearance, was normal in structure. (**b**) After 30 min, another ultrasonographic examination was performed after the completion of 20 min autonomous feeding. Because the gallbladder had contracted and become twisted in structure, it was impossible to view the region from the gallbladder base (arrowhead) to the cholecystic duct on the same screen. The measurements of the gallbladder were a long diameter of 10.9 mm, short diameter of 6.0 mm, and lumenal cross-sectional area of 0.27 cm^2^.

The cross-sectional area of the gallbladder lumen was reduced after feeding. The reduction ratio, calculated as a percentage of the cross-sectional area, was 49.5% (Figure 2).

**Figure 3:**
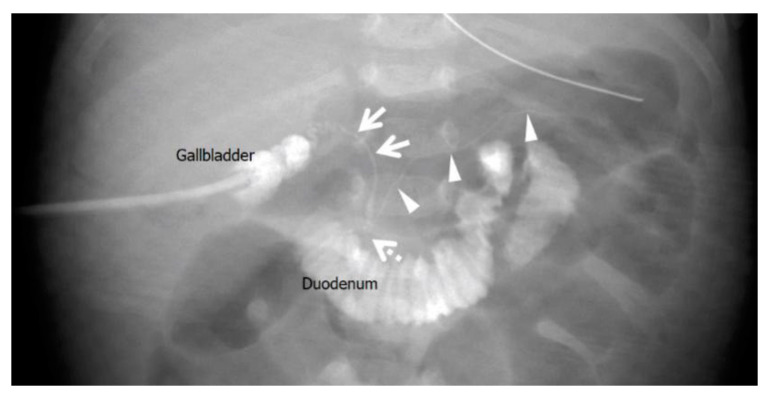
Intraoperative cholangiography in Case 1 at 30 days of age. The liver side was not contrast-enhanced through the common hepatic duct, and the duodenum was contrast-enhanced through the narrowed common bile duct (arrows). The pancreatic duct was also contrast-enhanced (arrowheads) and merged with the common bile duct in the pancreatic head to form a common channel (dotted arrow). The distance from the confluence to the duodenum was 5 mm, and a diagnosis of pancreaticobiliary maljunction with a common channel was made.

Biliary scintigraphy showed no bile excretion for up to 24 h. Magnetic resonance cholangiopancreatography showed an enlarged gallbladder lumen at 22 days of age, but no extrahepatic or intrahepatic bile ducts were delineated. Since US and MRCP could not rule out BA and biliary scintigraphy showed no excretion, we determined that intraoperative cholangiography was necessary to make a definitive diagnosis. Intraoperative cholangiography at 30 days of age did not depict the hepatic side of the common hepatic duct. However, it did show a common channel where the common bile and pancreatic ducts were opacified before passage of the contrast into the duodenum (Figure 3).

**Figure 4:**
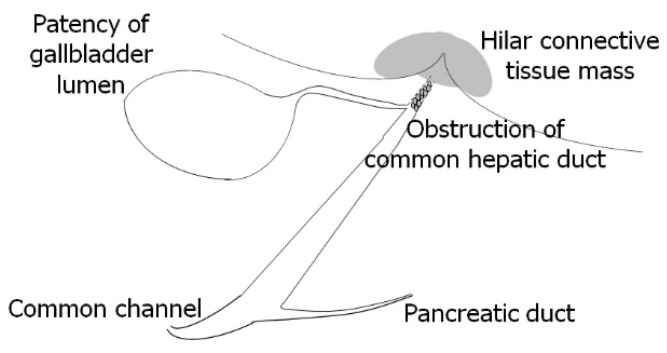
Surgical findings in Case 1 at 49 days of age during the Kasai procedure. The final diagnosis was type III biliary atresia according to the international classification of biliary atresia [5]: hilar obstruction, lower bile duct patency, and hilar connective tissue formation.

The Kasai procedure was performed at 49 days of age. The pancreatic amylase concentration in the bile within the gallbladder was 1 U/L (reference range, undetected), whereas the pancreatic amylase concentration in the serum was below the sensitivity of the measurement instrument. According to the international classification of BA, the final diagnosis was type III, denoting hilar obstruction and a patent lower bile duct [4] (Figure 4).

After surgery, the jaundice rapidly disappeared, and the liver dysfunction resolved.

### 2.2. Case 2: 67-Day-Old Girl

**Figure 5:**
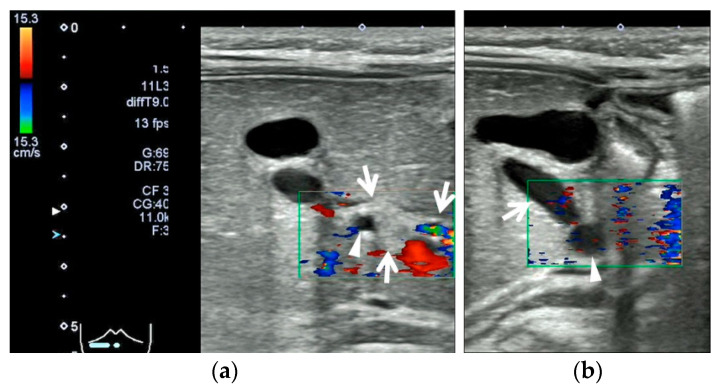
Abdominal ultrasonographic findings in Case 2 after 3 h of fasting; Aplio 300 (Cannon Medical Systems), linear probe, B-mode frequency: 9.0 MHz, pulse repetition frequency: 12 fps. (**a**) Transverse section in the right upper abdominal region: A common hepatic duct (arrowhead) was seen within a hyperechoic portal mass (enclosed by arrows). (**b**) Longitudinal section in the right upper abdominal region: The gallbladder lumen was open, and the gallbladder wall was normal in appearance (1.5 mm thick with a two-layered appearance). The choledochal duct was open (arrow), and the common hepatic duct (arrowhead) was tapered toward the hilar region. The maximum diameter and cross length of the gallbladder lumen were 11 × 11 mm.

The infant weighed 3100 g and required mechanical ventilation until day 5 after birth. She was brought to Japan at 47 days of age. At 49 days of age, she was seen by a local doctor for persistent jaundice and an umbilical hernia, and the jaundice was followed up. At 65 days of age, she developed grayish-white stools. At 67 days of age, she was seen again by her primary care physician, who suspected BA and referred her to our hospital for a follow-up visit. Blood and biochemical tests at the initial visit revealed cholestatic liver dysfunction (aspartate aminotransferase, 151 U/L (reference range, 21–64 U/L); alanine aminotransferase, 91 U/L (reference range, 12–50 U/L); total bilirubin, 77 mg/L (reference range, <20 mg/L); direct bilirubin, 58 mg/L (reference range, <3 mg/L); gamma-glutamyl transpeptidase, 221 U/L (reference range, 30–250 U/L); serum pancreatic amylase, 1 U/L (reference range, 1–6 U/L); and total bile acids, 242 µmol/L (reference range, <20 µmol/L)). Abdominal ultrasound showed the TCS and dilatation of the gallbladder lumen and cholecystic duct. The gallbladder wall was 1.5 mm thick and exhibited a two-layered appearance, and there was no evidence of gallbladder atrophy (Figure 5).

**Figure 6:**
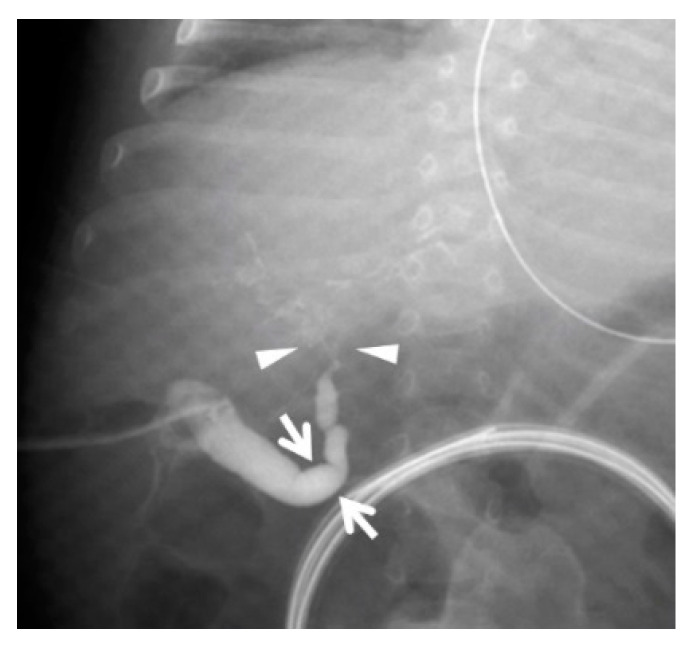
Intraoperative cholangiography in Case 2 at 73 days of age. The common bile duct was not contrast-enhanced, and the gallbladder and choledochal duct (arrows) were enlarged. The common hepatic duct narrowed toward the porta hepatis, and the intrahepatic bile duct at the porta hepatis showed a mica-like appearance (arrowheads). The arrows show the same site as the arrow in Figure 5b, and the arrowheads show the same area as that enclosed by arrows in Figure 5a.

There was no change in the cross-sectional area of the gallbladder lumen from before to after feeding. Biliary scintigraphy showed no bile excretion for up to 24 h. Magnetic resonance cholangiopancreatography showed luminal enlargement from the choledochal duct to the common hepatic duct; however, no intrahepatic bile ducts were shown because of a sudden narrowing at the porta hepatis, and the lower bile duct was not depicted. Since US and MRCP could not rule out BA and biliary scintigraphy showed no excretion, we determined that intraoperative cholangiography was necessary to make a definitive diagnosis. Intraoperative cholangiography at 73 days of age showed obstruction downstream of the confluence of the three ducts and stenosis at the porta hepatis. The intrahepatic bile duct was diffusely stenosed, and the hilar region had a mica-like appearance (Figure 6).

**Figure 7:**
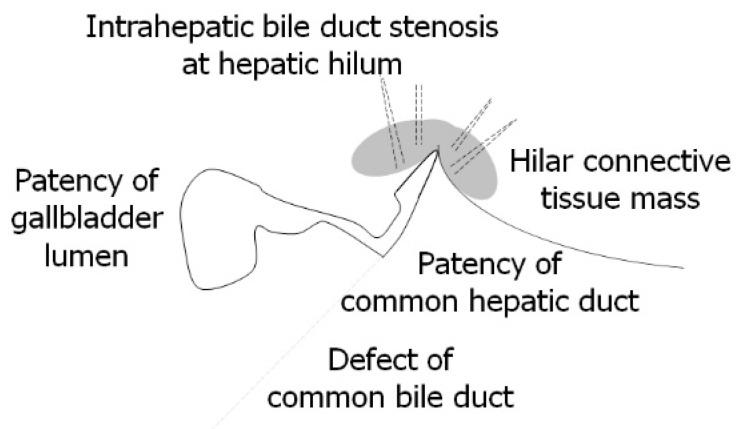
Surgical findings in Case 2 at 79 days during the Kasai procedure. The final diagnosis was type I biliary atresia according to the international classification of biliary atresia [5]: hilar bile duct patency, hilar connective tissue formation, and lower bile duct obstruction (defect).

The Kasai procedure was performed at 79 days of age. The concentration of pancreatic amylase in the bile juice within the gallbladder was below the sensitivity of the measurement instrument. According to the international classification of BA, the final diagnosis was type I, denoting a lower bile duct defect and a patent hilar bile duct [4] (Figure 7).

After surgery, the jaundice rapidly disappeared, and the liver dysfunction resolved.

## 3. Discussion

Ultrasonography is frequently the initial imaging test performed in the diagnosis of persistent neonatal jaundice, and it focuses on differentiating between BA and other disorders [6]. Typical ultrasonographic findings indicative of BA are the TCS, a small or unidentified gallbladder with a hazy wall, and the absence of contraction of the gallbladder after feeding [1,2,3]. However, a normal gallbladder with an open lumen is present in approximately 10% of cases [3].

Notably, despite the ultrasonographically normal gallbladder in both of our patients with BA, only the patient in Case 1 showed post-feeding gallbladder contraction. The difference between Cases 1 and 2 was the anatomical physiology of the lower bile duct. The patient in Case 1 had an open lower bile duct, whereas the patient in Case 2 did not.

In their study of the pathophysiology of BA complicated by PM in patients with a normal gallbladder and the TCS, Endo et al. [4] concluded that patency of the gallbladder lumen is due to pancreatic juice reflux and that the TCS is due to inflammation of the hilar region caused by pancreatic juice, which progresses over time. The presence of ductal and microcystic structures within the TCS suggests that the pancreatic amylase concentration in the bile within the gallbladder in Case 1 may have been the basis for inflammation due to the reflux of pancreatic juice at the porta hepatis. Furthermore, Deguchi et al. [7] analyzed 43 cases of BA and reported that of the 5 cases in which intraoperative cholangiography was performed, 3 were complicated by PM. But there may be a case selection bias in Deguchi’s result. A Pubmed search result (searching for complications of BA and PM) suggests that such complications are rare. The epidemiological results indicate that the primary pathogenic mechanism of BA is inflammatory obstruction, and viral infections, immune responses, genetic predisposition [8,9], and pancreatic reflux have also been reported as possible causes of inflammatory obstruction [7,9]. The grayish-white stools that developed at 65 days of age in Case 2 indicate that the bile duct was gradually obstructed by a condition that results in progressive narrowing, such as chronic inflammation. The lumenal patency of the gallbladder in Case 2 might have been brought about by bile via the narrowed hilar bile duct.

In the diagnosis of BA, both the sensitivity and specificity of the ultrasonographic findings of gallbladder morphology and post-feeding contraction are reportedly low [2]. Because 10% of BAs show an open gallbladder lumen [3] and the frequency of BA complicated by PM is high, we can assume that the proportion of BA associated with post-feeding contraction of the gallbladder is also high [7]. The ultrasonographic findings of the gallbladder in Cases 1 and 2 were not indicative of BA. Therefore, if ultrasound examination shows abnormal findings of the gallbladder, it would be reasonable to consider these findings as a reference in diagnosing BA.

## 4. Conclusions

We presented herein our experience with two cases of BA with a positive TCS but normal morphology of the gallbladder, one with the presence of post-feeding contraction of the gallbladder and the other without. The difference between the cases was the presence or absence of patency of the lower bile duct, including the complication of BA and PM. We conclude that gallbladder findings are not diagnostic for BA because in some types of BA, the gallbladder may be normal in morphology and even have the capacity for contraction after feeding.

## Data Availability

The data that support the findings of this study are available from the corresponding author upon reasonable request.
